# Diabetic Osteopenia by Decreased β-Catenin Signaling Is Partly Induced by Epigenetic Derepression of sFRP-4 Gene

**DOI:** 10.1371/journal.pone.0102797

**Published:** 2014-07-18

**Authors:** Kiyoshi Mori, Riko Kitazawa, Takeshi Kondo, Michiko Mori, Yasuhiro Hamada, Michiru Nishida, Yasuhiro Minami, Ryuma Haraguchi, Yutaka Takahashi, Sohei Kitazawa

**Affiliations:** 1 Department of Pathology, Division of Diagnostic Molecular Pathology, Kobe University Graduate School of Medicine, Kobe City, Japan; 2 Department of Pathology, National Hospital Organization, Osaka National Hospital, Hoenzaka, Chuo-ku, Osaka City, Japan; 3 Department of Molecular Pathology, Ehime University Graduate School of Medicine, Shitsukawa, Toon City, Ehime, Japan; 4 Department of Diagnostic Pathology, Ehime University Hospital, Shitsukawa, Toon City, Ehime, Japan; 5 Department of Legal Medicine, Kobe University Graduate School of Medicine, Kobe City, Japan; 6 Department of Therapeutic Nutrition, Institute of Health Bioscience, The University of Tokushima, Tokushima City, Japan; 7 Department of Physiology and Cell Biology, Kobe University Graduate School of Medicine, Kobe City, Japan; 8 Department of Internal Medicine, Kobe University Graduate School of Medicine, Kobe City, Japan; Northeast Ohio Medical University, United States of America

## Abstract

In diabetics, methylglyoxal (MG), a glucose-derived metabolite, plays a noxious role by inducing oxidative stress, which causes and exacerbates a series of complications including low-turnover osteoporosis. In the present study, while MG treatment of mouse bone marrow stroma-derived ST2 cells rapidly suppressed the expression of osteotrophic Wnt-targeted genes, including that of osteoprotegerin (OPG, a decoy receptor of the receptor activator of NF-kappaB ligand (RANKL)), it significantly enhanced that of secreted Frizzled-related protein 4 (sFRP-4, a soluble inhibitor of Wnts). On the assumption that upregulated sFRP-4 is a trigger that downregulates Wnt-related genes, we sought out the molecular mechanism whereby oxidative stress enhanced the sFRP-4 gene. Sodium bisulfite sequencing revealed that the sFRP-4 gene was highly methylated around the sFRP-4 gene basic promoter region, but was not altered by MG treatment. Electrophoretic gel motility shift assay showed that two continuous CpG loci located five bases upstream of the TATA-box were, when methylated, a target of methyl CpG binding protein 2 (MeCP2) that was sequestered upon induction of 8-hydroxy-2-deoxyguanosine, a biomarker of oxidative damage to DNA. These *in vitro* data suggest that MG-derived oxidative stress (not CpG demethylation) epigenetically and rapidly derepress sFRP-4 gene expression. We speculate that under persistent oxidative stress, as in diabetes and during aging, osteopenia and ultimately low-turnover osteoporosis become evident partly due to osteoblastic inactivation by suppressed Wnt signaling of mainly canonical pathways through the derepression of sFRP-4 gene expression.

## Introduction

Many diabetic complications are ultimately induced by oxidative stress through advanced glycation end-products (AGEs)[1], [2] derived from the accumulation of methylglyoxal (MG)[3], an intermediate metabolite of glucose that increases in the serum or the organs of diabetics [4]–[6]. It is well known that the diabetic condition evokes a state of low-turnover osteoporosis, characterized by a severe decrease in the rate of osteoblast/osteoid surface and bone mineral apposition and in reduced bone strength – diabetic osteopathy. Recent studies by our group showing that transgenic mice that overexpress thioredoxin-1 (a protein that acts as an antioxidant by facilitating the reduction of substrates through cysteine thiol-disulfide exchange) are resistant to streptozotocin-induced diabetic osteopenia [7] clearly demonstrate that oxidative stress plays a crucial role in the development of diabetic osteopenia. Since bone is composed of two types of cells – bone-forming osteoblasts and bone-resorbing osteoclasts – the net balance between these two cell types ultimately defines the rate of bone turnover and bone mass. Together with the fact that oxidative stress *per se* has little effect on the number and function of bone-resorbing osteoclasts either *in vivo* or *in vitro* [7], [8], diabetic osteopenia can, by and large, be regarded as a condition induced by impaired anabolic functions of osteoblasts through oxidative stress [9].

Osteoblast differentiation is, on the other hand, driven by numerous factors, among which Wnt proteins activate both canonical and non-canonical pathways (well-characterized and established pathways) responsible for directing mesenchymal stem cells to differentiate toward the osteoblastic linage. Consequently, the question is whether and how oxidative stress affects Wnt signaling in the osteoblastic cell linage. *In vitro* oxidative stress (exemplified by H_2_O_2_) alters the function of cultured osteoblastic precursors by blocking the bone-anabolic function of canonical Wnt signaling through the diversion of the downstream signaling pathways of β-catenin from the T cell factor (Tcf)- to Forkhead box O (FoxO)-mediated transcription [8]. Also, chronic oxidative stress attributed to alcohol intake alters canonical Wnt/β-catenin signaling through the upregulation of DKK1, an antagonist of the canonical Wnt pathway [10]. Wnt signaling is, however, directly or indirectly modulated by numerous regulatory molecules [11], and, other than the above-mentioned mechanisms affecting the canonical Wnt pathway, little is known about how oxidative stress modulates Wnt signaling.

In this study, with the use of microarray analysis, we comprehensively screened the gene expression profiles of ST2 cells, derived from a multipotent bone marrow stromal cell line, in the presence or absence of oxidative stress induced by MG treatment; one of the Wnt antagonists, a secreted Frizzled-related protein 4 (sFRP-4) for both canonical and non-canonical Wnt signaling, was found upregulated by oxidative stress. Here, we propose a novel mechanism by which diabetic oxidative stress reduces bone volume by impairing Wnt signaling through the derepression of sFRP4 gene expression.

## Materials and Methods

### Cell Line and Cell Culture

Mouse bone marrow stromal cell-line ST2 (RIKEN, Tsukuba, Japan) was cultured in α-MEM (Sigma, St. Louis, MO) supplemented with 10% FBS (Sigma), 100 µg/ml penicillin/streptomycin (ICN Biomedicals, Inc., Aurora, OH) and 100 µM methylglyoxal (MG), and maintained at 37°C in a humidified atmosphere with 5% CO_2_.

### Comprehensive DNA Microarray Analysis and Quantitative Real-Time Reverse Transcription PCR (Q real-time RT-PCR)

Total RNA was isolated from ST2 cells treated with or without 100 µM methylglyoxal (MG) or 1 µM of 5-aza-2′-deoxycytidine (5-aza-dC) by standard methods with the use of an RNeasy Protect Mini kit (Qiagen KK, Tokyo, Japan) according to the manufacturer’s instructions. DNA microarrays, called Filgen Array Mouse 32 K (Filgen Incorporated, Nagoya, Japan), with Mouse Genome Oligo Set ver. 3.0 oligonucleotides (Operon Biotechnologies, Huntsville, AL) were used. Hybridization and scanning processes were conducted by Filgen Incorporated. Scanned data were analyzed with the use of Microarray Data Analysis Tool version 1.0 software (Filgen Inc.). Raw microarray data were submitted to the Gene Expression Omnibus (GEO) microarray data archive (http://www.ncbi.nlm.nih.gov/geo/) at the NCBI (accession numbers GSE52475). To assess the mRNA expression of sFRP-4, OPG, RANKL, thioredoxin 1 (TXN-1), Runx2, osteopontin (OPN), bone morphogenetic protein (BMP) and activin membrane-bound inhibitor (BAMBI), peroxisome proliferator activated receptor gamma (PPARgamma), adipocyte protein 2 (aP2), p16^INK4a^, Caspase 3 (Casp-3), and glyceraldehyde-3-phosphate dehydrogenase (GAPDH), 1 mg of total RNA was reverse-transcribed to synthesize cDNA that was then amplified and quantified by the ABI PRISM 7300 Real Time PCR system (Applied Biosystems, Foster City, CA) using a set of primers and probes (assay ID; sFRP-4, Mm00840104 ml; OPG, Mm00435452_m1; RANKL, Mm00441908_m1; TXN-1, Mm00726847_s1; Runx2, Mm00501578_m1; OPN, Mm00436767_m1; BAMBI, Mm00810458_s1; PPARγαµµα, Mm00440945_m1; aP2, Mm00445880_m1; p16^INK4a^, Mm00494449_m1; Casp-3, Mm00515916_m1; and GAPDH, No. 430813). mRNA expression was quantified relative to that of GAPDH in each reaction, according to the manufacturer’s protocol.

### Immunocytochemical Staining

ST2 cells treated with or without 100 µM of MG were fixed with 100% methanol and immunostained with the use of anti-mouse sFRP-4 antibody (ab32784; abcam, CA), counterstained with DAPI, and observed under a microscope.

### Mapping of Methylation Site by Sodium Bisulfite Modification

Genomic DNA was extracted from cultured ST2 cells, and the bisulfite reaction was carried out as previously described [12]. A quantity of 1 µg of DNA in a volume of 50 µl Tris-EDTA was denatured by NaOH (final concentration, 0.2 M) for 10 min at 37°C. Freshly prepared 30 µl of 10 mM hydroquinone and 520 µl of 3 M sodium bisulfite at pH 5 were added to the samples. Each sample was incubated under mineral oil at 50°C for 16 hr. Modified DNA was purified with Wizard DNA purification resin, according to the manufacturer’s recommended protocol (Promega, Madison, WI), and eluted into 50 µl of H_2_O. Modification was completed by NaOH (final concentration, 0.3 M) treatment for 5 min at room temperature, and then by ethanol precipitation. DNA was resuspended in 20 µl of H_2_O and used immediately, or aliquots thereof were stored at −20°C until use. Bisulfite modified DNA (100 ng) was amplified with nested PCR using the following sets of converted primers covering the CpG accumulated loci (−107/41),

5′-GTAGGAGAGGTTGGGGGTGGAGT-3′: sense.

5′-CTTTCCTACTAAAACTCCAATCTA-3′: antisense.

5′-GGGGTGGAGTAGYGGAGTGGGAG-3′: nested-sense.

5′-CCAACACTCAACCTCCAAAACAA-3′: nested-antisense.

The PCR condition was as follows: 30 cycles at 94°C for 30 sec, 60°C for 30 sec, 72°C for 30 sec, and the final elongation step for 5 min at 72°C. The PCR mixture contained 1x buffer (Takara, Tokyo, Japan) with 1.5 mM of MgCl_2_, 20 pmol of each primer, 0.2 mM deoxynucleotide triphosphate, and bisulfite-modified DNA (50 ng) in a final volume of 50 µl. Each PCR product was loaded onto a 3% agarose gel, and the purified DNA from the gel was cloned into the pCR 2.1 plasmid vector (Invitrogen, Groningen, The Netherlands). After the transformation of and culturing the competent bacteria (INVαλπηαF’; Invitorgen) overnight on an LB/agar/ampicillin plate, colonies (at least 12) were randomly selected, and the recombinant plasmid was recovered for DNA sequencing with an M13F or M13R primer. The sequencing reactions for the cloned PCR products were carried out with a DNA sequencing kit (Applied Biosystems) and the reaction products were analyzed on a 310 Genetic Analyzer (Applied Biosystems). In all molecules examined, all cytosine residues not preceding guanine residues were converted by bisulfite treatment. Bisulfite treatment, PCR, cloning and sequencing analysis of DNA were repeated independently at least three times.

### Electrophoretic Mobility Shift Assay (EMSA)

Ready-to-use nuclear protein extracts of human cervical cancer cell line HeLa were purchased from Promega. For EMSA, double-stranded oligonucleotides composed of several combinations of the following sense- and antisense probes spanning TATA-box in the mouse *sFRP-4* basic promoter region (−56/−22) were used: unmethylated sense probe (UUS): 5′-GTCAGGGGACGCGTCTGGATAAATAGGGTTCCACA-3′, unmethylated antisense probe (UUA): 5′-TGTGGAACCCTATTTATCCAGACGCGTCCCCTGAC-3′, single-methylated sense probe (UMS): 5′-GTCAGGGGACG[5Me-dC]GTCTGGATAAATAGGGTTCCACA-3′, single-methylated antisense probe (UMA): 5′-TGTGGAACCCTATTTATCCAGA[5Me-dC]GCGTCCCCTGAC-3′, single-methylated sense probe (MUS): 5′-GTCAGGGGA[5Me-dC]GCGTCTGGATAAATAGGGTTCCACA-3′, single-methylated antisense probe (MUA): 5′-TGTGGAACCCTATTTATCCAGACG[5Me-dC]GTCCCCTGAC-3′, double-methylated sense probe (MMS): 5′-GTCAGGGGA[5Me-dC]G[5Me-dC]GTCTGGATAAATAGGGTTCCACA-3′, double-methylated antisense probe (MMA): 5′-TGTGGAACCCTATTTATCCAGA[5Me-dC]G[5Me-dC]GTCCCCTGAC-3′, and hydroxylated as well as single-methylated antisense probe (UOxMA): 5′-TGTGGAACCCTATTTATCCAGA[5Me-dC][8Ox-dG]CGTCCCCTGAC-3′ (Operon Biotechnologies). The oligonucleotides were annealed and 5′-end labeled with gamma^32^P –ATP (3,000 Ci/mmol) by T4 polynucleotide kinase (Promega). The binding reaction was carried out by preincubating labeled oligonucleotides with nuclear extracts at room temperature for 15 min. Specific antibodies against TATA-box binding protein (TBP) (ab818; Abcam, Cambridge, MA) and methylcytocine binding protein 2 (MeCP2) (ab2828) were added to the binding reaction for 45 min at 4°C. For the competition assay, 100-fold excess molar amounts of unlabeled double-stranded oligonucleotides (UUS/USA), containing the TATA-box sequence, were added to the binding reaction. Samples were electrophoresed on a 5% polyacrylamide gel at room temperature for 3 hr at 100 V; the gel was then dried and analyzed with image analyzer BAS-EWS 4075 (FUJIX, Tokyo, Japan).

### Chromatin Immunoprecipitation (ChIP) Assay

According to the instructions in the Acetyl-Histone H3/H4 Immunoprecipitation Assay Kit (Upstate Biotechnology, Lake Placid, NY), soluble chromatin was prepared from 1×10^6^ ST2 cells fixed with formaldehyde, precipitated with salmon sperm DNA/protein A agarose-50% slurry, and then 2 ml of the supernatant solution was incubated with an anti-TBP (ab818) or anti-MeCP2 antibody (ab2828) overnight at 4°C with rotation. The immunoprecipitate was collected after treatment with salmon sperm DNA/protein A agarose 50% slurry for 1 hr at 4°C with rotation, and then washed. From the eluted immunoprecipitate, DNA was recovered by phenol/chloroform extraction and ethanol precipitation, and resuspended with 1x Tris-EDTA buffer. PCR with the above DNA solution as a template was conducted using the following sets of primers covering the basic promoter region of mouse *sFRP-4* (−51/29),

5′-GGGACGCGTCTGGATAAATAGG-3′ (sense).

5′-AAGCTCCAGTCTCGAGTCCCGCAG-3′ (antisense).

### Western Blotting

ST2 cells treated with 100 µM of MG for 0 min (control), 30 min, 1 hr, 2 hr, 6 hr, 12 hr and 24 hr were collected. Aliquots of each cell lysate were separated by 10% sodium dodecyl sulfate (SDS)-polyacrylamide gel electrophoresis, and transferred onto a nylon membrane with the use of a semidry transfer system. The blotted membranes were incubated with a series of antibodies against signal transducing proteins related to canonical and non-canonical Wnt signaling (Wnt5a (AF645; R&D Systems, MN), Ror1 (4102; Cell Signaling Rec., MA), Ror2 [13], [14], p54 and p46 SAPK/JNK (9258; Cell Signaling Tec., MA), phospho-p54 and phospho-p46 SAPK/JNK (4668; Cell Signaling Tec., MA), c-jun (9165; Cell Signaling Tec., MA), phospho-c-jun (2361; Cell Signaling Tec., MA), β-catenin (9582; Cell Signaling Tec., MA) and phospho-β-catenin (2009; Cell Signaling Tec., MA)). The demonstrated bands were analyzed with NIH ImageJ for quantification.

### Statistical Analysis

Data are expressed as means ± SD. Statistical analyses were carried out by Student’s t-test. The level of significance was taken to be *p<0.05.*

## Results

### Gene Expression Profiles in Methylglyoxal-treated Cells

Of the 31,769 mouse genes displayed on the microchip, 475 demonstrated alteration in the level of expression: 354 were upregulated (more than 2-fold increase) whereas 121 were downregulated (more than 0.5-fold decrease). The former included genes encoding cluster of differentiation 34 (CD34), collagenase 3 precursor (MMP13), Gap junction alpha1 protein (connexin 43), adiponectin precursor, Jun D, glutathione S-transferase, and sFRP-4; the latter contained tumor necrosis factor receptor superfamily 11b (OPG), connective tissue growth factor precursor (CTGF), Wnt-1 inducible signaling pathway protein 1 (WISP1), G1/S-specific cyclin D1, tumor necrosis factor receptor associated factor 3 (TRAF3), WISP2, and integrin alphav precursor (Table 1).

**Table 1:** Genes showing upregulated (>2-fold increase) or downregulated (0.5-fold decrease) mRNA expression by 48-hr methylglyoxal treatment.

Up-regulated genes (extract)		Down-regulated genes (extract)	
Genes	Fold change	Genes	Fold change
Hematopoietic progenitor cell antigen (CD34)	9.473	Plasminogen activator inhibitor-1 precursor	0.137
Collagenase-3 precursor (Mmp13)	5.936	**Tumor necrosis factor receptor superfamily 11b (OPG)**	**0.206**
Adiponectin precursor	5.547	Connective tissue growth factor precursor (CTGF)	0.331
Cathepsin R precursor	4.769	Matrix Gla-protein precursor (MGP)	0.343
**Gap junction alpha-1 protein (Connexin 43)**	**4.138**	**Wnt-1 inducible signaling pathway protein-1 (WISP1)**	**0.387**
Cyclin-dependent kinase inhibitor-1 (p21)	3.352	**G1/S-specific cyclin D1**	**0.389**
Hepatocyte growth factor (HGF)	2.962	Tumor necrosis factor receptor associated factor 0–3 (TRAF3)	0.427
Growth arrest and DNA-damage-inducible 45 alpha (GADD45a)	2.615	**Wnt-1 inducible pathway signaling protein-2 (WISP2)**	**0.433**
**Transcription factor jun-D**	**2.565**	Mitogen-activated protein kinase kinase kinase-8 (MAPKKK8)	0.439
Rho family GTPase-1 (Rnd1)	2.561	Procollagen type XIX alpha-1 (COL19a1)	0.443
**Delta-like protein-3 precursor**	**2.524**	Cyclic AMP-dependent protein kinase inhibitor alpha (PIKA)	0.469
Collagen type XII alpha-1 precursor	2.295	Integrin alpha-II precursor	0.470
Collagen type VI alpha-1 precursor	2.165	Integrin alpha-V precursor	0.486
Glutathione S-transferase mu-4	2.162	ATP-binding cassette sub-family A	0.494
Transcription factor GATA-4	2.149	Tropomyosin-4	0.495
CBF1 interacting corepressor	2.126	Bcl-2 modifying factor	0.495
Histone H4	2.078		
**Secreted Frizzled-related protein-4 (sFRP-4)**	**2.044**		
GTP-binding protein-1	2.016		

Gene names written in bold letters indicate Wnt/β-catenin signaling pathway-related genes.

### Confirmation of Selected Gene Expression Using Q real-time RT-PCR

**Figure 1:**
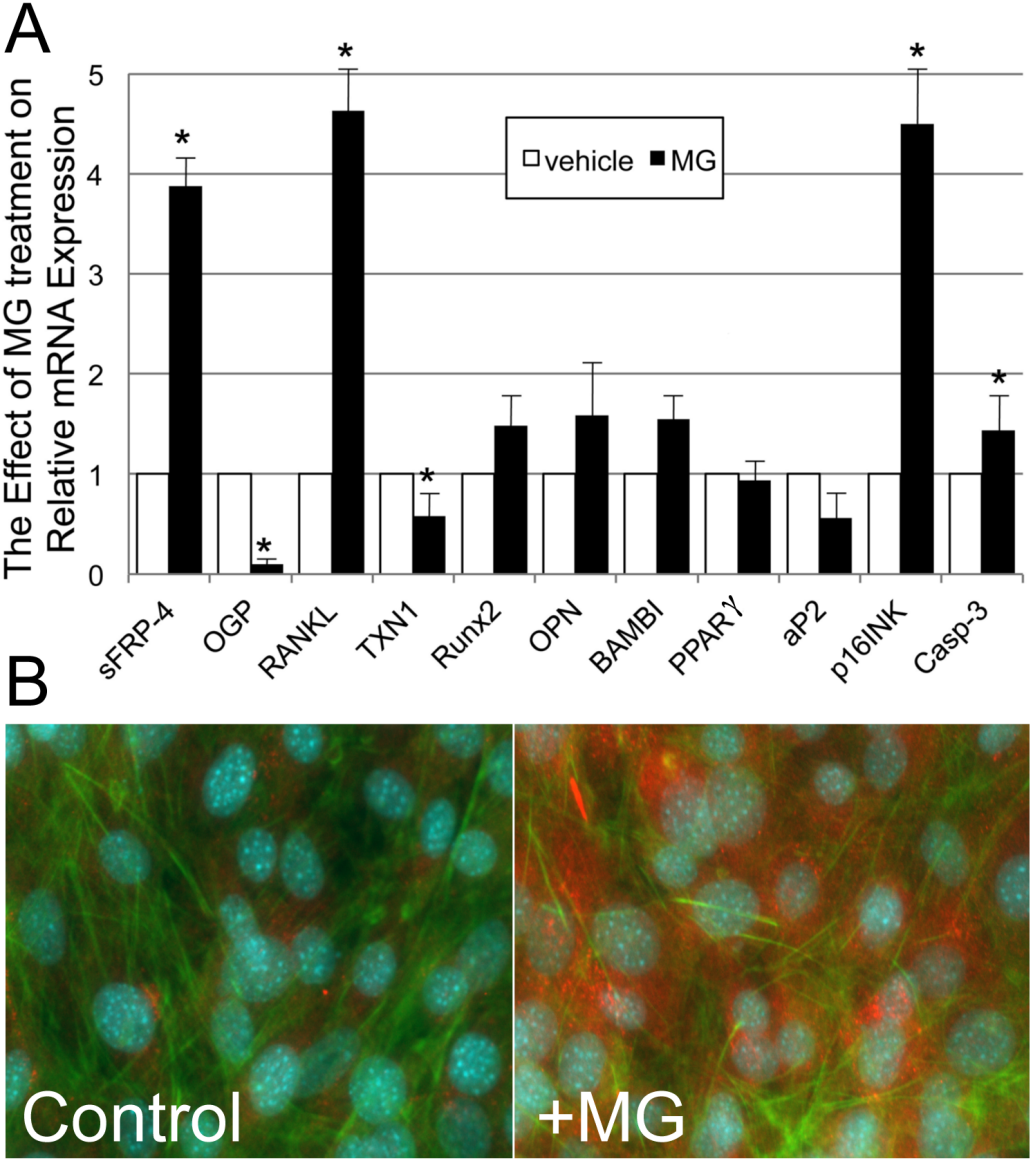
Confirmation of the effect of methylglyoxal on the expression of selected genes. (A) The effect of methylglyoxal (MG) treatment on the mRNA expression of sFRP-4, OPG, RANKL, Trx1, Runx2, OPN, BAMBI, PPARgamma, aP2, p16^INK4a^, and Casp-3 in ST2 cells. Administration of 100 µM of MG resulted in a 3.88- and a 4.63-fold increase in sFRP-4 and RANKL mRNA expression, respectively, but a decrease in OPG expression to 10% of its basal level. Quantitative real-time reverse transcription PCR (Q-real-time RT-PCR) confirmed reciprocal change in the expression levels between sFRP-4 and OPG. Furthermore, MG treatment increased p16^INK4a^ and caspase 3 expression, leading to cell cycle arrest and apoptosis, while markers of osteoblastic differentiation (Runx2, BAMBI, and OPN) increased slightly, and those for adipocytic differentiation (PPARγαµµα and aP2) decreased. The statistical significance was determined by Student’s t test, *P<0.05. (B) *In vitro* MG treatment for 12 hr, a model for the acute phase of oxidative stress on stromal cells, showed rapid induction of sFRP-4 protein in the cytoplasm of ST2 cells (right, x200, counterstained with phalloidin and DAPI), while little sFRP-4 protein was observed in the control (left, x200, counterstained with phalloidin and DAPI).

To confirm the reproducibility of the effect of MG-derived oxidative stress on the altered gene expression as demonstrated by the comprehensive microarray analysis, we quantified the mRNA level of sFRP-4 and OPG and, at the same time, tested the expression level of RANKL, Thioredoxin 1 (Trx1, disulfide oxidoreductase), several osteoblastic or adipocytic differentiation markers, cell cycle protein (p16^INK4a^), and apoptosis executioner Caspase 3 (Casp-3) in the cultured ST2 cells. Q real-time RT-PCR confirmed reciprocal change in the expression levels between sFRP-4 and OPG. Administration of 100 µM of MG resulted in a 3.88- and a 4.63-fold increase in sFRP-4 and RANKL mRNA expression, respectively. On the other hand, MG treatment decreased the expression of OPG to 10% of its basal level but upregulated that of p16^INK4a^ and Casp-3. By contrast, the expression levels of osteoblastic differentiation markers (Runx2, BAMBI, and OPN) increased, but not significantly, and those of adipocytic differentiation (PPARgamma and aP2) decreased modestly at most, indicating that short-term MG-derived oxidative stress did not affect the differential commitment of ST2 cells (Fig. 1A).

### Immunocytochemical Staining

*In vitro* MG treatment for 12 hr showed rapid induction of sFRP-4 protein in the cytoplasm of ST2 cells, while no sFRP-4 protein was observed in the control (Fig. 1B).

### Methylation Status of CpG Loci within Mouse sFRP-4 Gene Basic Promoter Region

**Figure 2:**
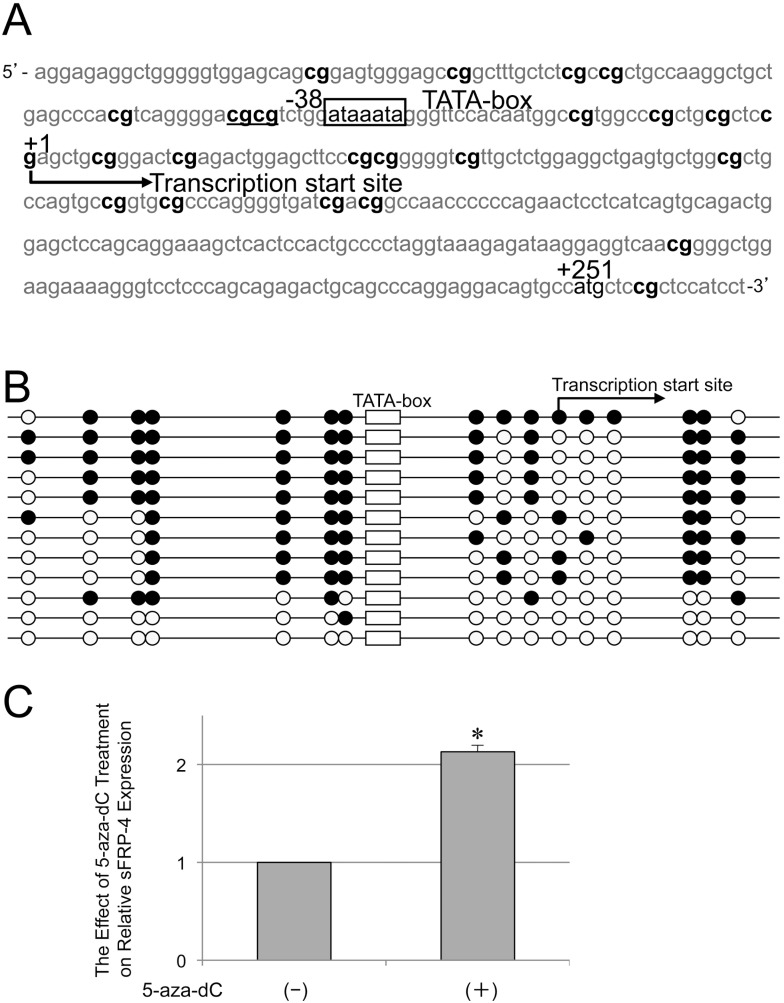
Status of CpG methylation in mouse sFRP-4 gene promoter region. (A) The basic promoter region of the sFRP-4 gene has TATA-box located 38 bases upstream of the transcription start site, around which CpG dinucleotides are clustered (shown in boldface). (B) Twelve independent bisulfite mappings. Corresponding to the repression of the *sFRP-4* gene expression in steady-state ST2 cells, CpGs within the mouse *sFRP-4* gene basic promoter, especially upstream of TATA-box, are highly methylated. (C) The effect of 2 nM of 5-aza-dC treatment for 72 hr on steady-state *sFRP-4* expression. Although not markedly prominent, quantitative RT-PCR showed that the steady-state expression of *sFRP-4* was, albeit two-fold at most, significantly restored by 5-aza-dC treatment. The statistical significance was determined by Student’s t test, *P<0.05.

Assessment of the 5′-flanking sequence of the mouse sFRP-4 gene frequently revealed clusters of CpG loci distributed around TATA-box 37 (Auth, cf. legend Fig. 2) bases upstream of the putative transcription start site (+1). Furthermore, two tandem CpGs were found five bases further upstream of TATA-box (Fig. 2A). Bisulfite mapping with the use of 12 independently cultured ST2 cells frequently revealed that the nearest two CpGs within the 5′-flanking region of TATA-box were methylated (20/24) (Fig. 2B), forming a target sequence of MeCP2 [15]. Moreover, Q real-time RT-PCR using total RNA extracted from ST2 cells cultured with 5-aza-2′-deoxycytidine (5-aza-dC) showed a 2.13-fold increase in sFRP-4 mRNA expression compared with its basal level (Fig. 2C). Long-term treatment of ST2 cells with MG showed that the methylation status of CpG loci around the basic promoter was not affected in either young (passage 6, P6) or old (passage 35, P35) ST2 cells (Fig. S1).

### Effect of Methylation and Oxidation of CpG Loci Five Bases Upstream of TATA-Box on MeCP2 Binding

**Figure 3:**
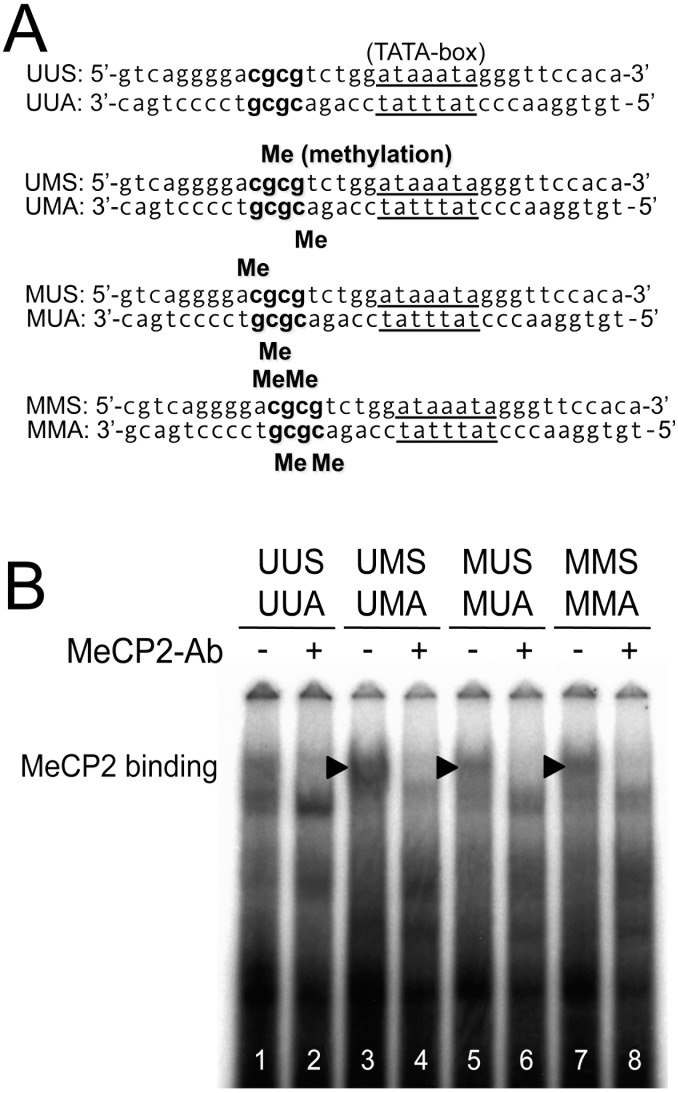
Effect of methylation at two CpG loci upstream of TATA-box on MeCP2 binding. *In vitro* binding of nuclear protein from HeLa cells to TATA-box and two CpG dinucleotides upstream of TATA-box was tested by EMSA. (A) Double-stranded oligonucleotides, unmethylated (UUS/UUA), single-methylated (UMS/UMA or MUS/MUA), and double-methylated (MMS/MMA) were subjected to the binding reaction. (B) Both single-methylated and double-methylated oligonucleotides showed prominent protein-DNA binding at a high position (black arrowheads, lanes 3, 5, and 7), which was block-shifted by the anti-MeCP2 antibody (lanes 4, 6, and 8). The sequence date has been quoted from GenBank (accession number AF364906.1) by Wong VK *et al* [41].

**Figure 4:**
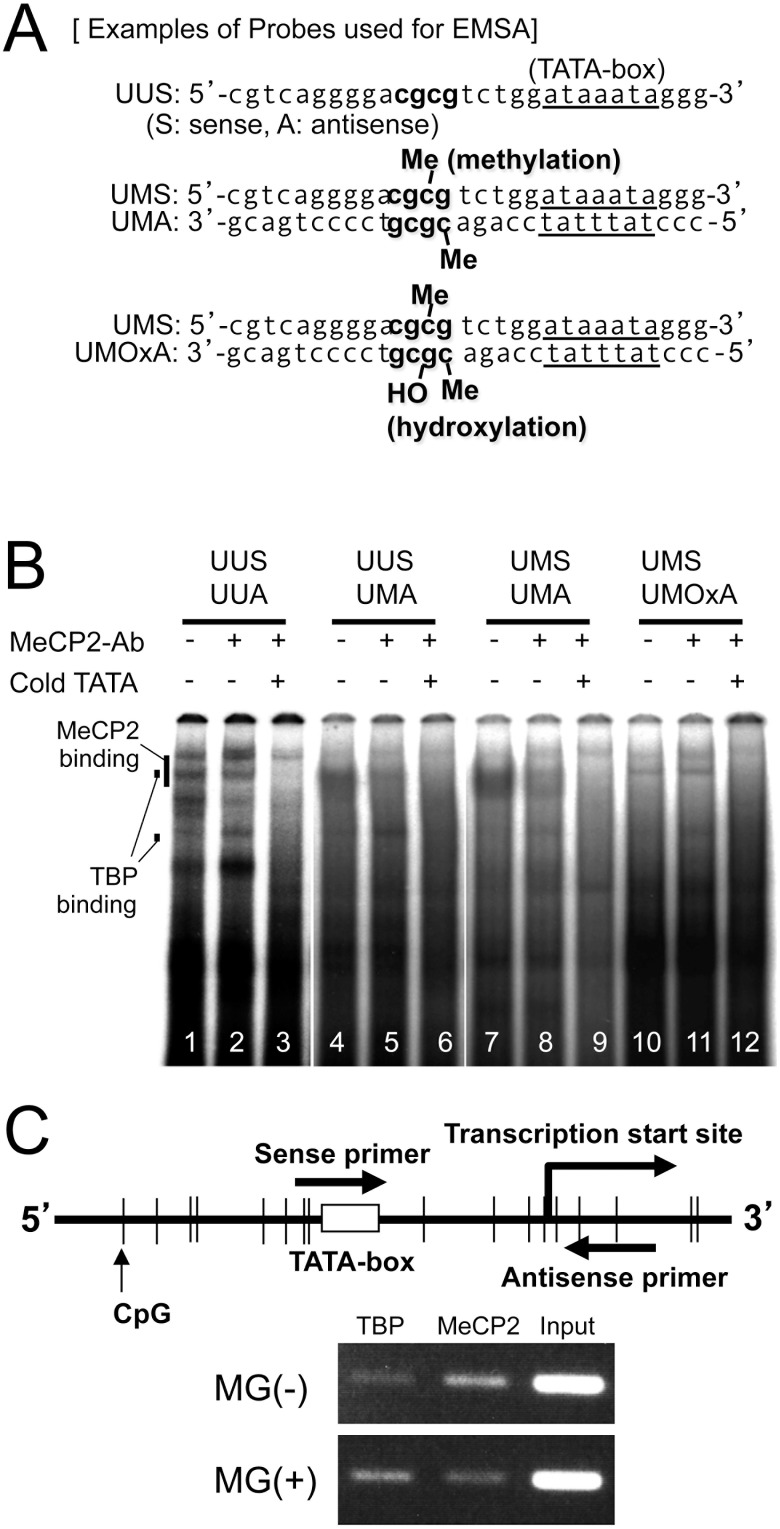
The effect of methylation and oxidation at CpG locus 5 bases upstream of TATA-box on MeCP2 binding. *In vitro* binding of nuclear protein from HeLa cells to TATA-box and CpGs 5 bases upstream of TATA-box was tested by EMSA. (A) Double-stranded oligonucleotides, unmethylated (UUS/UUA), hemimethylated (UUS/UMA), single-methylated (UMS/UMA), and hemihydroxilated as well as single-methylated (UMS/UOxMA) ones, spanning part of the mouse *sFRP-4* gene basic promoter region including TATA-box (−57/−29), were subjected to the binding reaction. (B) The unmethylated oligonucleotides show protein-DNA bindings (lanes 1 and 2, TBP binding) which are washed out by the cold consensus TATA-box sequence (lane 3). On the other hand, both hemi- and bi-methylated oligonucleotides show dense and clear protein-DNA binding (lanes 4 and 7, MeCP2 binding) which is block-shifted with an anti-MeCP2 antibody (lanes 5 and 8). At the same time, the protein-DNA bindings appearing at low and high positions (lanes 5 and 8, TBP binding) are washed out by a cold TATA-box competitor (lanes 6 and 9). By inducing a single 8-OHdG, protein-DNA bindings seen in lanes 4 and 7 have disappeared (lane 10), and alternative protein-DNA binding appearing at low and high positions (lanes 10 and 11, TBP binding) are washed out with a cold TATA-box competitor (lane 12). (C) A set of primers was used to amplify 80 bases of DNA containing a CpG dinucleotide, TATA-box and a transcription start site for ChIP assay of TBP and MeCP2 on the mouse sFRP-4 gene promoter. ST2 cells with or without MG treatment were subjected to immunoprecipitation. Input and immunoprecipitated DNA with either anti-TBP or anti-MeCP2 antibody was assessed by PCR using the set of primers as described above. Without MG treatment, a reactive PCR product reflecting mainly endogenous MeCP2 binding is observed. Conversely, a PCR product reflecting TBP binding was found mainly in MG-treated ST2 cells.

The effect of introducing single- or double-CpG(s) methylation five bases upstream of TATA-box on MeCP2 binding, as analyzed by EMSA, showed a marked difference between methylated and unmethylated probes. Double-stranded oligonucleotides, unmethylated (UUS/UUA), single-methylated (UMS/UMA or MUS/MUA), and double-methylated (MMS/MMA) were subjected to the binding reaction (Fig. 3A). Both single-methylated and double-methylated oligonucleotides showed prominent protein-DNA binding at a high position (Fig. 3B, lanes 3, 5, and 7), which was block-shifted by the anti-MeCP2 antibody (Fig. 3B, lanes 4, 6, and 8). Unmethylated sense and antisense oligonucleotides (UUS/UUA, Fig. 4A) showed several protein-DNA bindings, two of which were absorbed by excess of consensus TATA-box sequence (Fig. 4B, lane 3). On the other hand, both the hemi-(UUS/UMA, Fig. 4A) and bi-methylated (UMS/UMA, Fig. 4A) oligonucleotides showed prominent DNA-protein complexes (MeCP2 binding, Fig. 4B, lanes 4 and 7), which were block-shifted in the presence of the antibody specific for MeCP2 (lanes 5 and 8). At the same time, mirroring the decrease of MeCP2 binding in lanes 5 and 8, the TBP binding restored in these lanes was nullified by the excess of consensus TATA-box sequence (lanes 6 and 9). By inducing a single 8-OHdG (UMA/UMOxA, Fig. 4A), the protein-DNA binding (MeCp2 binding) seen in lanes 4 and 7 disappeared (lane 10), while the protein-DNA binding appearing at low and high positions (TBP binding) in lanes 10 and 11 was washed out with a cold TATA-box competitor (lane 12).

### Effect of Methylglyoxal (MG) Treatment on MeCP2 and TBP Binding with sFRP-4 Gene Basic Promoter Region

ST2 cells with or without MG treatment were subjected to ChIP assay using either an anti-MeCP2 or an anti-TBP antibody to amplify 80 bases spanning −51/+29 containing a CpG dinucleotide, TATA-box, and a transcription start site. A reactive PCR product reflecting endogenous MeCP2 binding was observed mainly in the nuclear precipitate from vehicle-treated ST2 cells. Conversely, a PCR product reflecting TBP binding was detected mainly in MG treated ST2 cells, confirming that oxidative stress attenuated MeCP2 binding with methylated CpGs and reciprocally promoted TBP binding to TATA-box at the cellular level (Fig. 4C).

### Western Blotting

**Figure 5:**
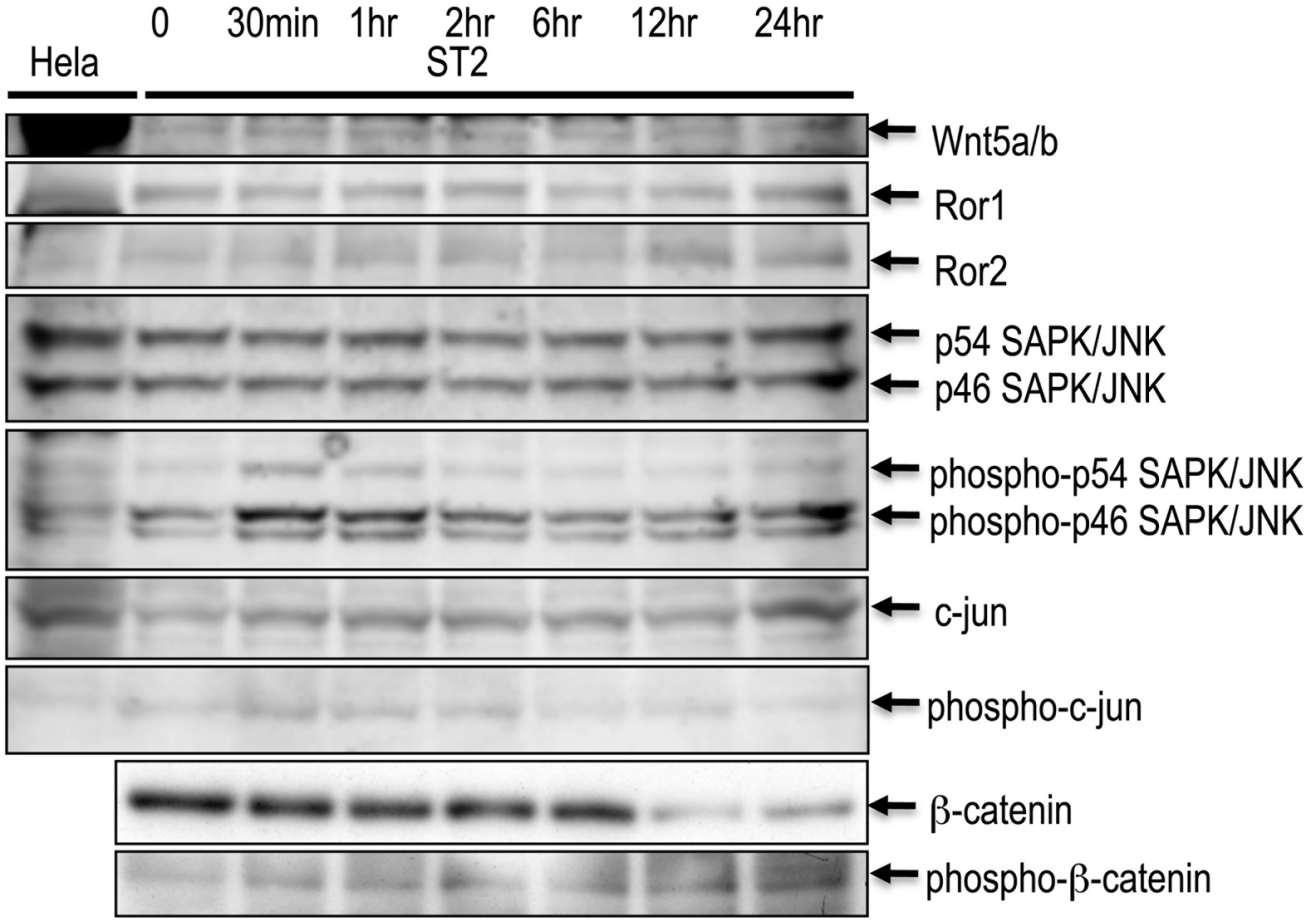
Western blotting for proteins related to canonical and non-canonical Wnt-signaling. ST2 cells with serial periods of MG treatment were subjected to Western blotting. Hela cell lysate was used as a control. While Wnt5a/b, Ror1, Ror2, whole- and phospho-SAPK/JNK, whole- and phospho-c-jun (factors related to non-canonical Wnt-signaling) were almost constant, the β-catenin protein level decreased and the phospho-β-catenin protein level increased reciprocally at 12 hr after treatment.

ST2 cells with serial periods of MG treatment were subjected to Western blotting with the use of Hela cell lysate as a control. While Wnt-signaling related to the non-canonical pathway (Wnt5a/b, Ror1, Ror2, whole- and phospho-SAPK/JNK, whole- and phospho-c-jun) was almost constant except for a transient increase (185% from control) of phospho-p46 (Fig. 5, 30 min to 2 hr), the β-catenin protein level decreased (28% from control) and the phospho-β-catenin protein level increased (135% from control) reciprocally at 12 hr after treatment (Fig. 5; β-catenin and phospho-β-catenin).

## Discussion

Bone formation and resorption are strictly regulated by two types of cells, osteoblasts and osteoclasts [16], [17]. Various bone-seeking inter- and intracellular signals including hormones, cytokines, enzymes, minerals, and transcription factors are known to modulate bone mass by controlling the proliferation, differentiation, and function of these two types of cells. Osteoblasts are specialized bone-matrix producing cells originating from undifferentiated skeletal mesenchymal cells [18]. Differentiation of osteoblastic cells is regulated by BMP-Smad and Wnt associated pathways (both canonical and non-canonical), followed by the induction of osteoblast-specific gene expression [19], [20]. Indeed, loss-of-function mutation in low-density lipoprotein receptor-related protein (Lrp) 5, a co-receptor for transduction of canonical Wnt signaling, leads to a low bone mass phenotype in both humans [21] and mice [22], whereas gain-of-function mutation in Lrp5 results in high bone mass in humans [22]–[25].

Several lines of evidence have shown that oxidative stress, which is closely associated with osteoporosis, decreases the function of osteoblasts [8], [26]–[28] and, conversely, increases the bone-resorbing activity of osteoclasts [29], [30]. Indeed, in our present *in vitro* study, many of the downregulated genes were Wnt/β-catenin signaling pathway-related; at the same time, the expression of RANKL, a factor essential for osteoclastogenesis, was upregulated (Table 1). We also noticed that sFRP-4, one of the secreted Frizzled-related proteins [31], was upregulated by oxidative stress. Since sFRP-4 is an antagonist of Wnt signaling, we speculated that the downregulation of Wnt signaling is, at least in part, attributable to upregulated sFRP-4 gene expression. We therefore directed our attention to the molecular mechanism whereby the sFRP-4 gene was upregulated by oxidative stress.

Oxidative stress has been shown to suppress the function and differentiation of osteoblastic cells by shifting intracellular Wnt/β-catenin signaling transducing factor Tcf-1 to Foxo-1 [8]. Although the link between oxidative stress and the regulation of sFRP-4 expression has not been reported to date, sFRP-4 gene expression has been shown as controlled by an epigenetic mechanism, especially by cytosine methylation, during cancer progression [32], [33]. CpG loci are, however, not concentrated around the promoter region of the sFRP-4 gene, and the frequently observed methylation determined by methylation**-**specific PCR is much lower than that of other sFRPs [34], [35]. In our study, base-level bisulfite mapping of methylated cytosine frequently revealed that two continuous CpG-loci upstream of TATA-box in the basic promoter region of the sFRP-4 gene were methylated in ST2 cells (Fig. 3B). We also noticed that the particular sequence of cgcgtctggataaata around TATA-box contained a typical MeCP2 target region (A/T sequences adjacent to methyl-CpG [15]). Moreover, the frequently observed methylation was not altered after long-term treatment of ST2 cells with MG (Fig. S1). Taken together, these results led to our speculation that the presence of a unique epigenetic silencing mechanism of the sFRP-4 gene is achieved by MeCP2 binding to TATA-box through the MeCP2 target region [15]. Indeed, in our current *in vitro* study, MeCP2 preferentially bound to oligonucleotides containing at least one methyl-CpG in two continuous CpG loci five nucleotides upstream of TATA-box (Fig. S2 and 4B). Our hypothesis was also supported by the ChIP assay, which showed that in sFRP-4 silenced ST2 cells, TBP binding was less prominent than MeCP2 binding to the sFRP-4 gene promoter region with methylated CpG loci (Fig. 4C). This unique epigenetic silencing mechanism is, in fact, functionally involved in the silencing of mouse RANKL gene by sequestering TBP [36].

We then raised the question of how oxidative stress derepresses epigenetically silenced sFRP-4 gene. Once CpG-loci scattered around the gene promoter are methylated, the reactivation process usually requires either de novo active demethylation [37] or passive demethylation through at least two continuous cell divisions without maintenance methylation. Since the methylated CpG was not affected by long-term treatment with MG (Fig. S1), demethylation, either passive or active, apparently was not involved in sFRP-4 reactivation. We therefore sought out an alternative epigenetic mechanism whereby MeCP2-binding to methylated CpG sites is inhibited by oxidative stress, and found that selective modification of guanine to 8-OHdG at CpG sites inhibits the binding of MeCP2 to its target sequences [38]. Consequently, we hypothesized that derepression of the sFRP-4 gene by oxidative stress is achieved by the inhibition of MeCP2 binding to the TATA-box area through the modification of 8-OHdG at the CpG site, followed by the restoration of TBP binding to TATA-box. Our *in vitro* binding study proved that induction of 8-OHdG at a CpG locus upstream of TATA-box completely blocks the binding of MeCP2 to its target sequence and, consequently, restores the binding of TBP to TATA-box (Fig. 4B, lanes 10–12). The ChIP assay also showed reciprocal binding between TBP and MeCP2 in the presence or absence of MG treatment (Fig. 4C). Although modification of guanine by oxidative stress is usually promptly cleared by base-excision repair [39], [40], such epigenetic alteration can be temporary and the presence of continuous oxidative stress, as in diabetic patients, may contribute to the initial and significant activation of the sFRP-4 gene, leading to immediate bone loss (Fig. S2). Moreover, because sFRP-4 is a soluble factor, it can be a good therapeutic target with the use of a neutralizing antibody or blocking agent.

**Figure 6:**
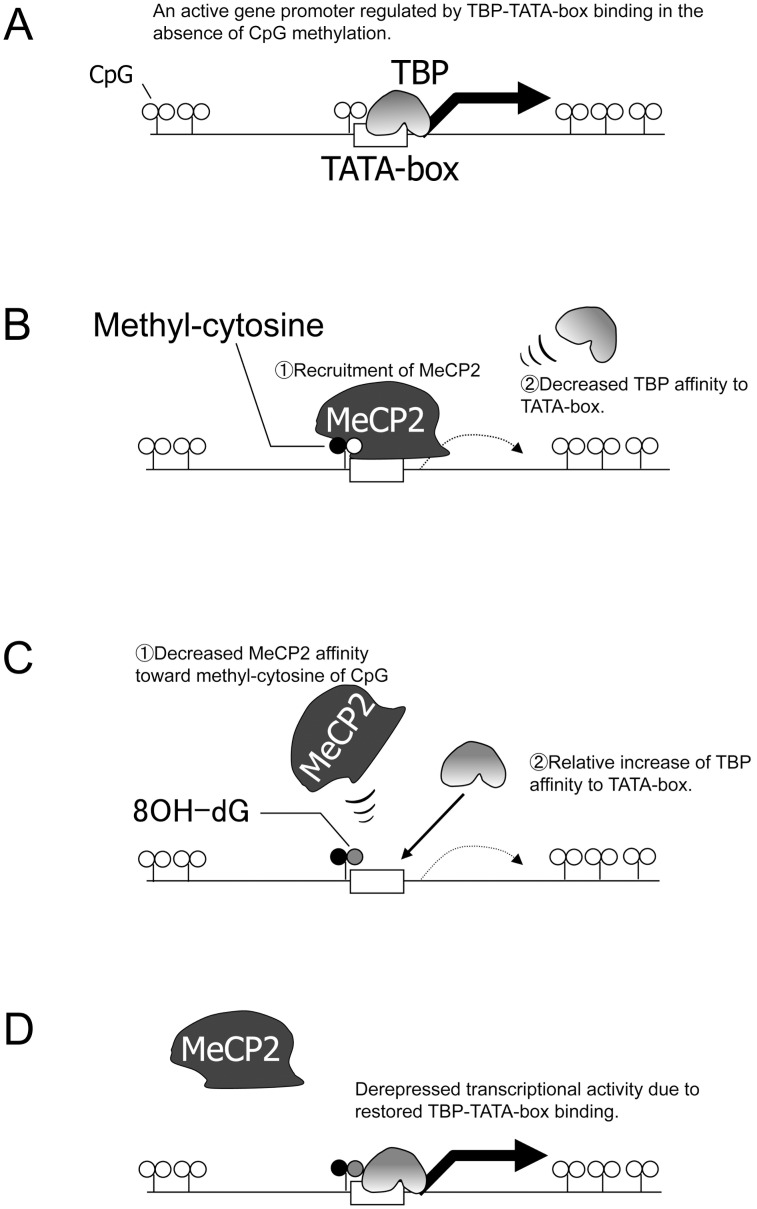
A schematic model of gene activation mechanism through 8-OHdG modification at methylated CpG locus upstream of TATA-box. When the CpG locus is not methylated, TBP gains access to TATA-box and transactivates the gene. (B) By the addition of single methylation at the CpG locus, the sequence becomes the target of MeCP2. As a result, TBP binding to TATA-box is disturbed, and the gene becomes inactive. (C) Guanine modification by oxidative stress typically results in the induction of hydroxylation at position 8, which sequesters MeCP2 binding to the target DNA. (D) As a consequence, TBP binding to TATA-box is restored, and the gene is reactivated.

In conclusion, we propose a unique epigenetic model in which modified guanine attributed to oxidative stress derepresses the sFRP-4 gene by sequestering MeCP2 and conversely recruiting TBP at TATA-box (Fig. 6).

## Supporting Information

Figure S1 **Methylation status of CpG loci around sFRP-4 basic promoter in ST2 cells (passage 6, P6; and passage 35, P35) after continuous MG treatment.** *In vitro* continuous MG treatment did not affect the methylation status of CpG loci around the sFRP-4 basic promoter in young (P6) and old (P35) ST2 cells.
(TIF)

Figure S2 **Possible mechanism of bone loss due to oxidative stress.** Oxidative stress strongly represses OPG gene expression through the activation of sFRP-4 expression prior to the inhibited Wnt/β-catenin signal transduction. Furthermore, in the chronic phase of oxidative stress, the persistently inhibited Wnt/β-catenin signal may lead to the arrest of osteoblast differentiation that would negatively affect both bone formation and resorption, and may ultimately induce low-turnover osteoporosis.
(TIF)
