# Supplementary figures and images for: Diabetic Osteopenia by Decreased β-Catenin Signaling Is Partly Induced by Epigenetic Derepression of sFRP-4 Gene
Source: PLoS One. 2014 Jul 18;9(7):e102797. doi: 10.1371/journal.pone.0102797 (PMC4103869; doi:10.1371/journal.pone.0102797)

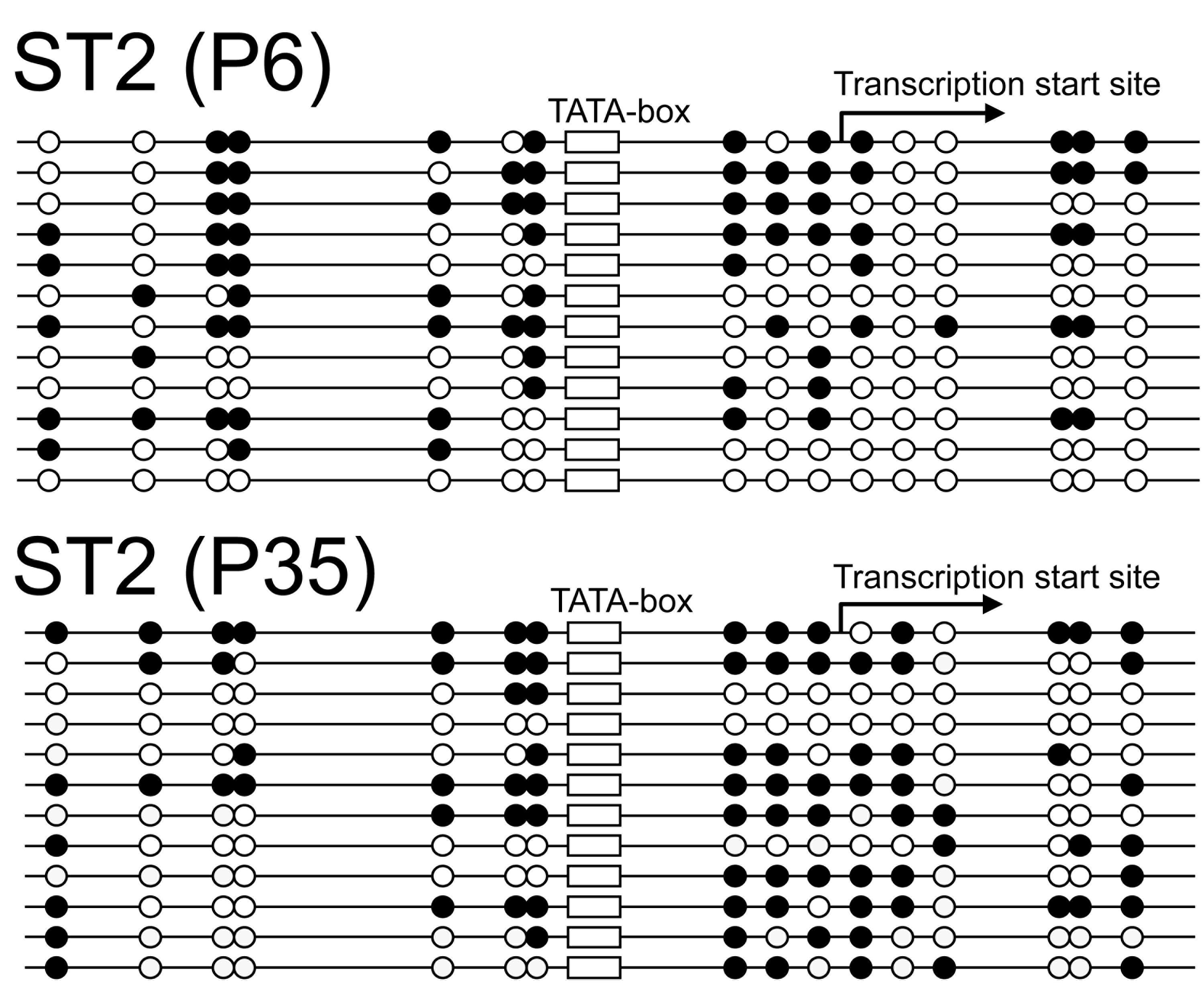

Supplement: Figure S1 — Methylation status of CpG loci around sFRP-4 basic promoter in ST2 cells (passage 6, P6; and passage 35, P35) after continuous MG treatment. In vitro continuous MG treatment did not affect the methylation status of CpG loci around the sFRP-4 basic promoter in young (P6) and old (P35) ST2 cells. (TIF) [file pone.0102797.s001.tif]

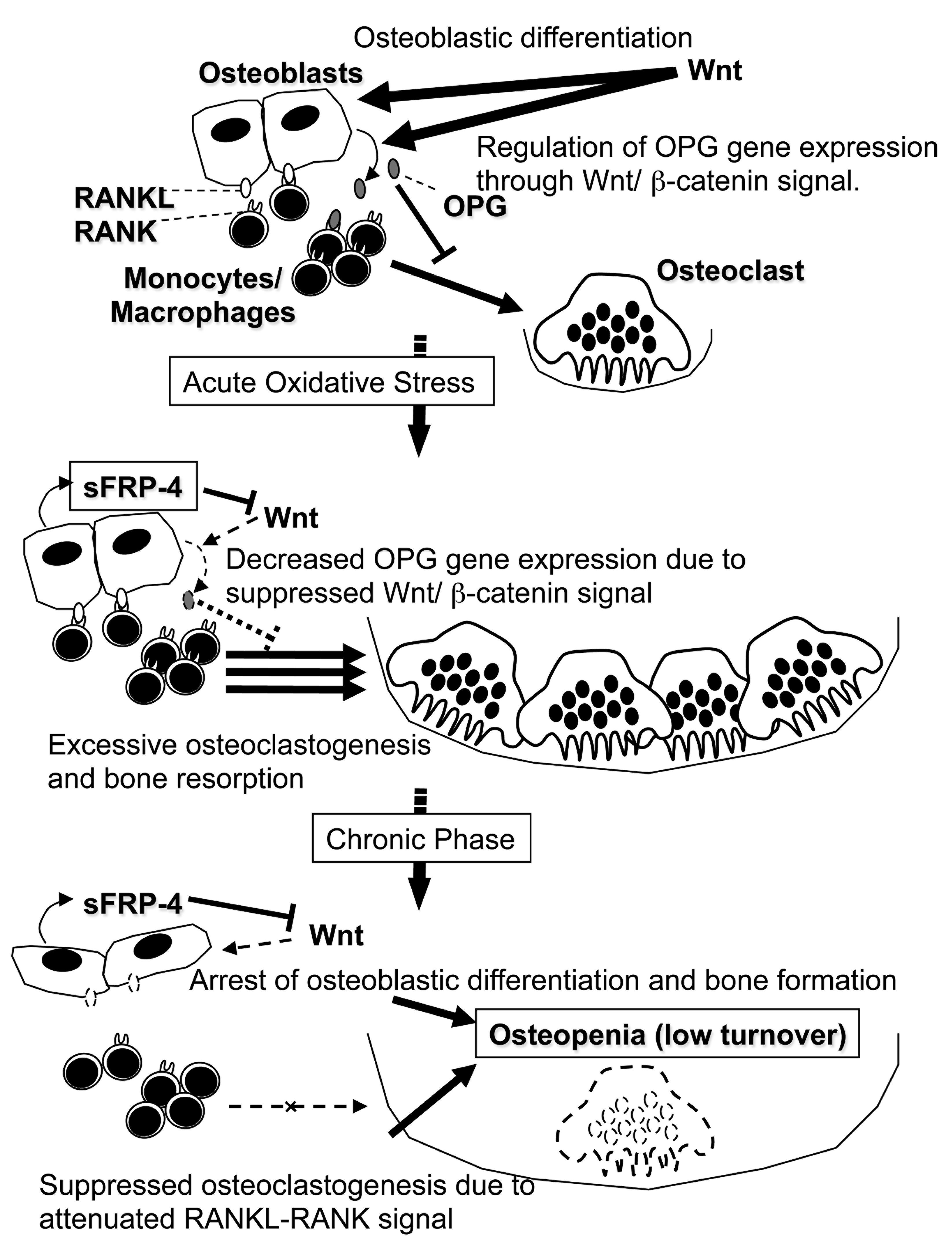

Supplement: Figure S2 — Possible mechanism of bone loss due to oxidative stress. Oxidative stress strongly represses OPG gene expression through the activation of sFRP-4 expression prior to the inhibited Wnt/β-catenin signal transduction. Furthermore, in the chronic phase of oxidative stress, the persistently inhibited Wnt/β-catenin signal may lead to the arrest of osteoblast differentiation that would negatively affect both bone formation and resorption, and may ultimately induce low-turnover osteoporosis. (TIF) [file pone.0102797.s002.tif]
